# Long-term changes and effect of pterygium size on corneal topographic irregularity after recurrent pterygium surgery

**DOI:** 10.1038/s41598-020-65376-4

**Published:** 2020-05-21

**Authors:** Takashi Ono, Yosai Mori, Ryohei Nejima, Jinhee Lee, Kentaro Abe, Yuji Nagata, Takuya Iwasaki, Makoto Aihara, Kazunori Miyata

**Affiliations:** 1 Miyata Eye Hospital, Miyazaki, Japan; 2 Department of Ophthalmology, Graduate School of Medicine, The University of Tokyo, Tokyo, Japan

**Keywords:** Eye diseases, Conjunctival diseases, Corneal diseases, Eye abnormalities, Refractive errors, Vision disorders

## Abstract

This retrospective observational study compared long-term topographic changes after recurrent- and primary-pterygium surgery depending on pterygium size. Patients who underwent recurrent-pterygium excision between 2002–2013 and age, sex, and pterygium size-matched controls who underwent primary-pterygium surgery were included (33 eyes of 33 patients in each group). Pterygium size was graded per advancing edge position: <1/3 of corneal diameter (grade 1), outside the pupil (grade 2), and within the pupillary area (grade 3). Surface asymmetry index (SAI), surface regularity index (SRI) in corneal topography, and uncorrected and best-spectacle-corrected visual acuity were compared before and 1, 3, 6, and 12 months postoperatively. Three, 17, and 13 eyes had grades 1, 2, and 3, respectively. In grade 2, the SAI and SRI were respectively significantly larger at all observation points (p = 0.01, 0.03, 0.02, 0.02, and 0.004) and before and 6 and 12 months postoperatively (p = 0.02, 0.04, and 0.03) in recurrent pterygium. In grade 3, the SAI was significantly larger before and 1, 3, and 12 months postoperatively (p = 0.04, 0.01, 0.01, and 0.02) and the SRI was significantly larger before and 12 months postoperatively (p < 0.001, 0.02) in recurrent pterygium. Corneal irregularity persisted 12 months after recurrent-pterygium surgery compared with that in same-size primary pterygium.

## Introduction

Pterygium is a wing-shaped proliferative disease of the conjunctival and the subconjunctival tissues invading the cornea. Its prevalence is reported at 10.2% and occurs in patients ranging widely in age based on a systematic review^1^. Several surgical techniques are used for its treatment such as bare sclera^2^, primary closure^3^, conjunctival autograft^4^, limbal conjunctival autograft^5^, conjunctival flap^6^, and amniotic membrane graft^3^, while their long-term efficacy is under debate. Corneal irregularity is an important consideration in maintaining good visual acuity and visual function after pterygium surgery and it does not improve early postoperatively; restoration of the corneal surface requires a long time, and Nejima *et al*. have reported that 6 to 12 months are needed to attain stability of corneal irregularity for larger sized pterygium^7^. They have also demonstrated that the restoration of corneal topographic changes after surgery of the primary pterygium depended on pterygium size^7^.

One of the clinical problems of pterygium surgery is recurrence, and with a relatively high rate considering the prevalence of this disease and the numbers of patients undergoing surgery^8^. One of the challenges of pterygium surgery is degradation of the Bowman’s layer and invasiveness^9^, making it difficult to separate proliferative tissue from the corneal stroma, with the difficulty being more pronounced in recurrent-pterygium surgery. Therefore, it was hypothesised that corneal irregularity and time-course changes after recurrent-pterygium surgery would differ from those after primary-pterygium surgery. Indeed, it was reported that corneal aberration was larger in a recurrent-pterygium than in a primary-pterygium group 12 months postoperatively^10^. Although the size of the pterygium correlates with corneal irregularity and visual function^11^, the effect of different recurrent-pterygium sizes on corneal irregularity has not been sufficiently elucidated. Evaluation of visual function is clinically important to decide whether an operation should be performed. Additionally, the time needed to restore corneal sphericity and irregularity is important because patients may need to acquire spectacles after surgery or they may require cataract surgery. Therefore, it is clinically necessary to estimate the postoperative period required for corneal surface to completely stabilise. In this study, we compared the long-term topographic changes after surgery for recurrent and primary pterygium depending on pterygium size.

## Subjects and methods

This was a retrospective observational study. It was reviewed and approved by the Institutional Review Board of Miyata Eye Hospital (Miyazaki, Japan). All study procedures adhered to the tenets of the Declaration of Helsinki. All subjects provided informed consent.

We included consecutive eyes that underwent excision of recurrent pterygium at Miyata Eye Hospital from January 2002 to December 2013 and were observed for more than 12 months for corneal irregularity evaluation. We excluded eyes that had undergone previous operations for pterygium more than twice or excision of pterygium of both the nasal and temporal sides. Age, sex, and pterygium size-matched patients who underwent primary-pterygium surgery at Miyata Eye Hospital between January 2002 and December 2015 served as controls. The pterygium size was graded according to the advancing edge position based on a previous report^7,12^: less than one third of the corneal diameter (grade 1), outside the pupil (grade 2), and within the pupillary area (grade 3).

Regarding the surgical techniques for recurrent pterygium, transplantation of the preserved limbal allograft and the amniotic membrane was performed after excision of the recurrent pterygium as described in previous reports^13^. All patients underwent identical operations. In brief, after topical anaesthesia, the pterygium head was removed from the cornea. The conjunctival and subconjunctival fibrovascular tissues were entirely removed. Scarring on the cornea was bluntly cleaned. Mitomycin (MMC) 0.04% was administered for 1 minute and irrigated. To cover the bare sclera, the amniotic membrane was sutured with a 10–0 polyglycolic acid suture (Alcon, Fort Worth, TX, USA). The limbal allograft was trimmed and sutured to cover the limbal deficient area with a 10–0 nylon suture (Alcon). These procedures were also followed for primary-pterygium surgery; after irrigation with MMC, the adjacent superior or inferior conjunctiva was moved on the bare sclera^14^. Topical 0.5% levofloxacin (Cravit, Santen, Osaka, Japan) and 0.1% betamethasone sodium phosphate (Rinderon, Shionogi, Osaka, Japan) four times a day were instilled for at least 3 months after the surgery. Topical 0.1% fluorometholone was started and tapered off after topical betamethasone use. Recurrence of pterygium was diagnosed when the proliferative tissue invaded within the cornea.

The values for uncorrected visual acuity (UCVA), best spectacle-corrected visual acuity (BSCVA), and corneal topography were retrospectively obtained from the medical records before and 1, 3, 6, and 12 months after the operation. Corneal topography was evaluated with a TMS-2 Corneal Topographer (Tomey, Nagoya, Japan). Refractive power, astigmatism, surface asymmetry index (SAI), surface regularity index (SRI), and the rate of changes in the SAI and SRI were compared between the recurrent- and primary-pterygium groups.

For statistical analysis, the Kruskal–Wallis test with Steel-Dwass test, Fisher’s exact t test, and Welch’s t test were used depending on data distribution. All values are presented as mean ± standard deviation. Statistical analyses were performed using the BellCurve for Excel (Social Survey Research Information, Tokyo, Japan) and the statistical significance was set at p < 0.05.

## Results

In total, 66 eyes of 66 patients were included in this study. Thirty-three eyes of 33 patients had recurrent pterygium and 33 eyes of 33 patients had primary pterygium. The mean age was 70.3 ± 7.4 years in the recurrent-pterygium and 69.4 ± 8.1 years in the primary-pterygium group. The numbers of eyes with grades 1, 2, and 3 in the primary and recurrent-pterygium groups were 3, 17, and 13, respectively. The characteristics of patients with each pterygium grade are shown in Table 1. In grade 1, there was no significant difference in the SAI, SRI, preoperative UCVA, preoperative BSCVA, corneal astigmatism, and corneal refractive power between the two groups. In grade 2, the SAI and SRI were larger in the recurrent-pterygium than in the primary-pterygium group (p = 0.01 and 0.02, respectively). In grade 3, the SAI and SRI were larger in the recurrent-pterygium than in the primary-pterygium group (p = 0.04 and <0.001, respectively). There were no significant differences in the other examined factors between the primary- and recurrent-pterygium groups in grades 2 and 3. The respective recurrence rates in the primary- and recurrent-pterygium groups were 0% and 0% in grade 1, 5.9% and 5.9% in grade 2, and 0% and 7.7% in grade 3, without any significant difference between the two groups.

**Table 1:** Characteristics of patients with each pterygium size.

	Grade 1		p-value	Grade 2		p-value	Grade 3		p-value
	Primary	Recurrent		Primary	Recurrent		Primary	Recurrent	
N	3	3		17	17		13	13	
Sex (Male: Female)	1:2	1:2	1	8:9	8:9	1	5:8	5:8	1
Age (years)	74.3 ± 1.5	73.7 ± 1.5	0.62	69.1 ± 9.0	70.0 ± 8.8	0.51	68.7 ± 7.6	68.5 ± 7.6	0.96
Preoperative surface asymmetry index	0.25 ± 0.10	0.56 ± 0.25	0.15	0.60 ± 0.35	1.11 ± 0.69	0.01*	1.22 ± 0.72	1.84 ± 0.75	0.04*
Preoperative surface regularity index	0.16 ± 0.09	0.38 ± 0.20	0.19	0.52 ± 0.32	1.20 ± 1.01	0.02*	0.75 ± 0.49	2.75 ± 1.19	<0.001*
Preoperative uncorrected visual acuity (logMAR)	0.46 ± 0.47	0.34 ± 0.22	0.71	0.35 ± 0.29	0.42 ± 0.32	0.48	0.43 ± 0.55	0.64 ± 0.46	0.30
Preoperative best spectacle-corrected visual acuity (logMAR)	0.31 ± 0.60	−0.01 ± 0.21	0.44	0.06 ± 0.13	0.10 ± 0.24	0.51	0.20 ± 0.55	0.28 ± 0.24	0.66
Preoperative corneal astigmatism (D)	1.8 ± 1.3	0.8 ± 0.1	0.27	2.9 ± 1.7	3.3 ± 2.3	0.57	4.6 ± 1.5	5.1 ± 3.7	0.64
Preoperative corneal refractive power (D)	43.0 ± 1.8	44.6 ± 0.5	0.22	43.7 ± 1.0	44.2 ± 2.4	0.43	43.0 ± 1.6	40.4 ± 5.5	0.12

*p < 0.05.

logMAR: logarithm of the minimum angle of resolution, D: dioptres.

All values are presented as mean ± standard deviation.

In grade 1, the SAI was larger in the recurrent-pterygium group 1 month postoperatively (p = 0.03), but the other examined values were not significantly different at any observation point after the surgery (Table 2). The change rate in the SAI and SRI did not significantly differ between the two groups. Longitudinal change in the SAI and SRI in grade 1 was not significant in both the primary- and recurrent-pterygium groups.

**Table 2:** Surface asymmetry index (SAI), surface regularity index (SRI), and change rate of the SAI and SRI after grade 1 pterygium surgery.

	SAI		p-value	Change rate of the SAI (%)		p-value	SRI		p-value	Change rate of the SRI (%)		p-value
	Primary	Recurrent		Primary	Recurrent		Primary	Recurrent		Primary	Recurrent	
Pre	0.25 ± 0.10	0.56 ± 0.25	0.15	100	100	1	0.16 ± 0.09	0.38 ± 0.20	0.19	100	100	1
1 month	0.29 ± 0.08	1.00 ± 0.24	0.03*	118.8 ± 22.2	212.0 ± 120.3	0.31	0.25 ± 0.17	0.53 ± 0.27	0.22	143.2 ± 46.8	192.1 ± 157.5	0.65
3 months	0.37 ± 0.12	0.54 ± 0.30	0.56	153.2 ± 40.3	79.7 ± 13.5	0.07	0.30 ± 0.29	0.72 ± 0.58	0.48	154.4 ± 88.4	308.3 ± 363.0	0.66
6 months	0.29 ± 0.11	0.75 ± 0.28	0.09	142.3 ± 109.3	145.7 ± 75.9	0.97	0.11 ± 0.05	0.60 ± 0.27	0.08	87.7 ± 59.2	162.1 ± 14.9	0.15
12 months	0.20 ± 0.11	0.63 ± 0.53	0.30	79.8 ± 18.2	128.8 ± 139.8	0.61	0.15 ± 0.18	0.61 ± 0.43	0.19	119.5 ± 153.6	167.6 ± 117.2	0.69

*p < 0.05.

All values are presented as mean ± standard deviation.

**Figure 1:**
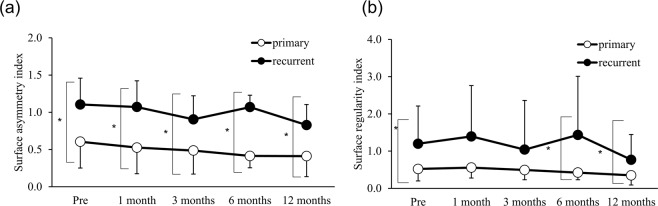
Surface asymmetry index (SAI) and surface regularity index (SRI) of grade 2 pterygium. (**a**) Preoperative and postoperative SAI of grade 2 pterygium. There was significant difference between primary and recurrent pterygium before and at 1, 3, 6, and 12 months after surgery (*p = 0.01, 0.03, 0.02, 0.02, and 0.004, respectively). Compared to the preoperative values, there was no significant difference at any observation point in both primary and recurrent pterygium. (**b**) Preoperative and postoperative SRI of grade 2 pterygium. There was significant difference between primary and recurrent pterygium before and at 6 and 12 months after surgery (*p = 0.02, 0.04, and 0.03, respectively). Compared to the preoperative values, there was no significant difference at any observation point in both primary and recurrent pterygium.

In grade 2, the SAI was significantly larger at all observation points (Fig. 1a; p = 0.01, 0.03, 0.02, 0.02, and 0.004, respectively) and the SRI was significantly larger before and 6 and 12 months after the operation (Fig. 1b; p = 0.02, 0.04, and 0.03, respectively) in the recurrent-pterygium group. Longitudinal change in the SAI and SRI in grade 2 was not significant in both the primary- and recurrent-pterygium groups. There was no difference in the change rate between the two groups (Table 3).

**Table 3:** Change rate of the surface asymmetry index (SAI) and surface regularity index (SRI) after surgery of grade 2 pterygium.

	Change rate of the SAI		p-value	Change rate of the SRI		p-value
	Primary	Recurrent		Primary	Recurrent	
Pre	100	100	1	100	100	1
1 month	117.8 ± 99.1	117.1 ± 88.9	1.00	188.6 ± 278.2	100.9 ± 65.0	0.23
3 months	100.8 ± 75.5	97.3 ± 46.0	0.88	168.7 ± 260.6	88.3 ± 57.4	0.23
6 months	113.1 ± 126.4	107.0 ± 66.1	0.86	147.6 ± 197.3	102.5 ± 69.3	0.39
12 months	92.3 ± 75.5	87.6 ± 45.0	0.83	92.0 ± 100.1	76.2 ± 40.3	0.55

All values are presented as mean ± standard deviation.

**Figure 2:**
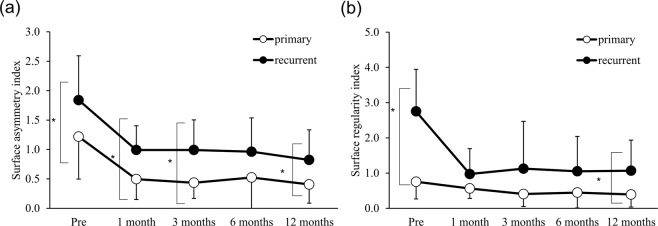
Surface asymmetry index (SAI) and surface regularity index (SRI) of grade 3 pterygium. (**a**) Preoperative and postoperative SAI of grade 3 pterygium. There was significant difference between primary and recurrent pterygium before and at 1, 3, and 12 months after surgery (*:p = 0.04, 0.01, 0.01, and 0.02, respectively). Compared to the preoperative value, the SAI in primary pterygium significantly decreased at 1, 3, 6, and 12 months (p = 0.005, 0.003, 0.007, and 0.003, respectively) and the SAI in recurrent pterygium significantly decreased at 1, 3, 6, and 12 months (0.01, 0.01, 0.01, and 0.004, respectively). (**b**) Preoperative and postoperative SRI of grade 3 pterygium. There was a significant difference between primary and recurrent pterygium before and at 12 months after surgery (*p < 0.001 and p = 0.02, respectively). Compared to the preoperative values, the SRI in primary pterygium significantly decreased at 3 months (p = 0.03) and the SRI in recurrent pterygium significantly decreased at 1, 3, 6, and 12 months (p = 0.005, 0.004, <0.001, and 0.002, respectively).

In grade 3, the SAI was significantly larger before and 1, 3, and 12 months postoperatively (Fig. 2a; p = 0.04, 0.01, 0.01, and 0.02, respectively) and the SRI was significantly larger before and 12 months after the operation (Fig. 2b; p < 0.001, p = 0.02, respectively) in the recurrent-pterygium group. Compared with the preoperative value, the SAI in the primary-pterygium group significantly decreased at 1, 3, 6, and 12 months (p = 0.005, 0.003, 0.007, and 0.003, respectively), and the SRI significantly decreased at 3 months (p = 0.03). Compared with the preoperative value, the SAI in the recurrent-pterygium group significantly decreased at 1, 3, 6, and 12 months (0.01, 0.01, 0.01, and 0.004, respectively) and the SRI significantly decreased at 1, 3, 6, and 12 months (p = 0.005, 0.004, <0.001, and <0.002, respectively). The SAI was 0.82 ± 0.51 and the SRI was 1.07 ± 0.87 12 months after recurrent-pterygium surgery, showing persistent corneal irregularity. There was no difference in the change rate between the two groups except at 1 month after the operation (Table 4; p = 0.01).

**Table 4:** Change rate of the surface asymmetry index (SAI) and surface regularity index (SRI) after surgery of grade 3 pterygium.

	Change rate of the SAI		p-value	Change rate of the SRI		p-value
	Primary	Recurrent		Primary	Recurrent	
Pre	100	100	1	100	100	1
1 month	45.7 ± 22.9	76.7 ± 42.9	0.07	107.6 ± 77.1	39.2 ± 17.7	0.01*
3 months	42.0 ± 24.1	60.1 ± 20.2	0.07	71.8 ± 51.0	38.1 ± 28.7	0.07
6 months	40.0 ± 21.3	58.5 ± 33.2	0.12	58.4 ± 44.7	43.9 ± 34.3	0.37
12 months	35.1 ± 21.4	58.0 ± 50.9	0.15	81.3 ± 92.2	39.8 ± 24.1	0.14

*p < 0.05.

All values are presented as mean ± standard deviation.

There was a significant preoperative difference in corneal irregularity between the pterygium grades: between grades 1 and 3 (p = 0.021) and grades 2 and 3 (p = 0.027) for the SAI and between grades 1 and 3 (p = 0.043) for the SRI in the primary-pterygium group; between grades 2 and 3 (p = 0.019) for the SAI and between grades 1 and 3 (p = 0.021) and grades 2 and 3 (p = 0.004) for the SRI in the recurrent-pterygium group. One month postoperatively, there was a significant difference between grades 1 and 2 for the SAI in the primary-pterygium group (p = 0.04), but no such difference was observed for the recurrent-pterygium group. Three months postoperatively, there were no grade differences between the primary and recurrent pterygiums. There was significant difference between grades 1 and 2 in SRI of the primary pterygium group 6 months postoperatively (p = 0.03). There were no grade differences between the primary and the recurrent-pterygium groups 12 months postoperatively.

The UCVA and BSCVA values of the two groups are summarised in Table 5. In grade 1, there was no significant difference between the two groups in UCVA and BSCVA. In grades 2 and 3, although there was no difference in UCVA, BSCVA was worse in the recurrent-pterygium group after 3, 6, and 12 months (0.04, 0.004, and 0.04 in grade 2 and 0.02, 0.03, and 0.01 in grade 3, respectively).

**Table 5:** Uncorrected and best-spectacle-corrected visual acuity of patients with each grade pterygium.

Uncorrected visual acuity (logMAR)		Grade1		p-value	Grade2		p-value	Grade3		p-value
		Primary	Recurrent		Primary	Recurrent		Primary	Recurrent	
	Pre	0.46 ± 0.47	0.34 ± 0.22	0.71	0.35 ± 0.29	0.42 ± 0.32	0.48	0.43 ± 0.55	0.64 ± 0.46	0.30
	1 month	0.41 ± 0.37	0.29 ± 0.24	0.67	0.35 ± 0.23	0.35 ± 0.25	0.98	0.34 ± 0.53	0.47 ± 0.44	0.54
	3 months	0.47 ± 0.42	0.20 ± 0.17	0.35	0.18 ± 0.29	0.33 ± 0.28	0.15	0.15 ± 0.22	0.41 ± 0.35	0.05
	6 months	0.44 ± 0.49	0.17 ± 0.16	0.42	0.18 ± 0.29	0.32 ± 0.30	0.17	0.18 ± 0.19	0.37 ± 0.32	0.10
	12 months	0.44 ± 0.49	0.22 ± 0.18	0.51	0.17 ± 0.28	0.28 ± 0.25	0.21	0.22 ± 0.17	0.33 ± 0.28	0.25
**Best-spectacle-corrected visual acuity (logMAR)**		**Grade1**			**Grade2**			**Grade3**		
		**Primary**	**Recurrent**	**p-value**	**Primary**	**Recurrent**	**p-value**	**Primary**	**Recurrent**	**p-value**
	Pre	0.31 ± 0.60	-0.01 ± 0.21	0.44	0.06 ± 0.13	0.10 ± 0.24	0.51	0.20 ± 0.55	0.28 ± 0.24	0.66
	1 month	0.19 ± 0.55	0.03 ± 0.18	0.65	0.00 ± 0.15	0.04 ± 0.21	0.49	0.09 ± 0.56	0.19 ± 0.26	0.57
	3 months	0.24 ± 0.41	-0.01 ± 0.17	0.39	-0.08 ± 0.07	0.03 ± 0.20	0.04*	-0.06 ± 0.09	0.10 ± 0.19	0.02*
	6 months	0.22 ± 0.53	-0.03 ± 0.14	0.49	-0.10 ± 0.09	0.06 ± 0.20	0.004*	-0.06 ± 0.09	0.08 ± 0.19	0.03*
	12 months	0.17 ± 0.46	-0.01 ± 0.15	0.54	-0.09 ± 0.10	0.00 ± 0.16	0.04*	-0.07 ± 0.09	0.11 ± 0.21	0.01*

*p < 0.05.

logMAR: logarithm of the minimum angle of resolution.

All values are presented as mean ± standard deviation.

## Discussion

Recurrent pterygium is reported to cause large postoperative corneal aberration^10^, however, long-term topographic change evaluation after pterygium surgery has not been reported in detail. The current study showed that corneal irregularity after surgery was larger in recurrent-pterygium than in the primary-pterygium group for the same pterygium grade. The SAI and SRI significantly decreased after surgery for recurrent pterygium invading inside the pupil area on the cornea (grade 3) but remained elevated 12 months postoperatively. The SRI is a useful topographic index to evaluate corneal astigmatism, and the SAI reflects corneal asymmetricity; values lower than 0.5 are considered normal^15–17^. It has been reported that large pterygium causes large regular and irregular astigmatism^18,19^, but even for pterygium of the same size, other factors, such as three-dimensional thickness, might cause further irregularity. In the current study, high corneal irregularity 12 months after recurrent-pterygium surgery, which could not be corrected with glasses, might contribute to BSCVA worsening in the recurrent-pterygium group. Conversely, the change rate in corneal irregularity remained near identical for 12 months postoperatively between the two groups. It was suggested that corneal morphological change after pterygium surgery was constant regardless of primary- or recurrent-pterygium surgery. As the preoperative corneal irregularity of the recurrent-pterygium group was large, the recurrent-pterygium group at grades 2 and 3 required more than 12 months for corneal topography restoration, which was longer than that required for the primary-pterygium group.

It has been suggested that there are differences with respect to the pathological nature between recurrent and primary pterygium. In general, pterygium connects strongly to the corneal epithelium and invades the corneal stroma beyond the Bowman’s layer. Garcia *et al*. reported that goblet cell density decreased over the surface of recurrent pterygium as observed with impression cytology and suggested that there is a difference between primary and recurrent pterygium^20^. These characteristics warrant deep surgical intervention into the corneal stroma and affect the postoperative roughness of the corneal stroma and epithelium.

Previous reports have shown that corneal wavefront aberrations were higher after recurrent pterygium surgery than after primary pterygium surgery and that the pterygium size correlated with the degree of corneal wavefront aberration^10^. However, the previous report included patients with various sizes of pterygium and did not compare corneal irregularity with the same pterygium size. Therefore, we clarified that recurrent pterygium caused large corneal irregularity with the same pterygium size. Recurrent pterygium appears more elevated than primary pterygium. It naturally progresses over the scars of primary pterygium, suggesting that there remains essential corneal irregularity caused by the surgery for primary pterygium, which would lead to additional roughness on the cornea after recurrent-pterygium surgery. The current study did not estimate the size of the primary pterygium that had been previously removed, but it may be related to the persistent irregularity. Furthermore, the differences in surgical techniques may have contributed to the current result. The current study included only patients with recurrent pterygium after transplantation of the preserved limbal allograft and amniotic membrane. Amniotic membrane transplantation with intraoperative MMC administration might effectively reduce the recurrence of pterygium and postoperative suppression of ocular inflammation could have an effect on early stability of the ocular surface^21^.

We compared the changes in corneal irregularity based on the size of the recurrent pterygium. For both primary and recurrent pterygium, preoperative irregularity increased proportionally to the pterygium size. The results of the primary-pterygium group showed that there was significant difference among grades even 6 months after the operation, supporting the findings of a previous report^7^. In contrast, there was no significant difference among the pterygium grades in the recurrent-pterygium group. Especially, grades 2 and 3 showed highly persistent corneal irregularity, decreasing visual function.

This study had several limitations. First, the number of grade 1 pterygium was very small because of the effect of grade 1 pterygium on visual function and the number of patients with indication for surgery. However, the caused corneal irregularity was small because of the small pterygium size (Table 1). Second, the mire ring used for the topographic change estimation could be affected by large pterygium size and lead to image defects. Further study evaluating the corneal topography with anterior segment optical coherence tomography is necessary. Third, although many surgical techniques are in use, we did not examine the effect of surgical technique on corneal irregularity, partly because of the retrospective nature of the study. Further prospective multicentre studies would be necessary to validate our findings.

Corneal irregularity was larger in the recurrent-pterygium than in the primary-pterygium group for the same pterygium size 12 months postoperatively. On the contrary, the change rate of corneal irregularity was almost identical for 12 months. Longer and thorough observation is necessary after recurrent-pterygium surgery.

### Financial disclosures

No author has a financial or proprietary interest in any material or method mentioned.

## Data Availability

The data that support the findings of this study are available on request from the corresponding author, T.O. The data are not publicly available due to their containing information that could compromise the privacy of research participants.
